# Quantum circuits with SINIS structures

**DOI:** 10.3762/bjnano.16.134

**Published:** 2025-11-04

**Authors:** Mikhail Tarasov, Mikhail Fominskii, Aleksandra Gunbina, Artem Krasilnikov, Maria Mansfeld, Dmitrii Kukushkin, Andrei Maruhno, Valeria Ievleva, Mikhail Strelkov, Daniil Zhogov, Konstantin Arutyunov, Vyacheslav Vdovin, Vladislav Stolyarov, Valerian Edelman

**Affiliations:** 1 V. Kotelnikov Institute of Radio Engineering and Electronics RAS, Moscow, Russia; 2 Institute of Applied Physics RAS, Nizhny Novgorod, Russia; 3 Special Astrophysical Observatory RAS, Nizhnii Arkhyz, Russia; 4 ITMO University, Saint Petersburg, Russia; 5 P.N. Lebedev Physical Institute of the Russian Academy of Sciences, 119991 Moscow; 6 National Research University "Higher School of Economics," Moscow, 101000 Russia; 7 P. Kapitza Institute for Physical Problems RAS, Moscow, Russia

**Keywords:** Big Telescope Alt-azimuthal, electron coolers, microwave detectors, micro- and nanotechnology, NIS tunnel junction, superconducting tunnel junctions

## Abstract

The superconductor–insulator–normal metal–insulator–superconductor (SINIS) tunnel junction structure is the basic building block for various cryogenic devices. Microwave detectors, electron coolers, primary thermometers, and Aharonov–Bohm interferometers have been fabricated by various methods and measured at temperatures down to 100 mK. The manufacturing methods included Dolan-type shadow evaporation, Manhattan-type shadow evaporation, and magnetron sputtering with selective etching of superconducting and normal metal electrodes. Improvement in ultimate sensitivity is achieved by suspending the absorber above the substrate. Best responsivity of up to 30 electrons per photon at a frequency of 350 GHz, or 72000 A/W, and voltage responsivity up to 3.9 × 10^9^ V/W were obtained with a black body radiation source and series of band-pass filters. The specially designed SINIS arrays are intended to detect 90 GHz radiation at the “Big Telescope Alt-azimuthal” (romanized Russian: “Bolshoi Teleskop Alt-azimutalnyi”, BTA) with noise equivalent power of less than 10^−16^ W·Hz^−1/2^. The receiver in a ^3^He cryostat with an optical window was mounted at the Nasmyth focus of the BTA and tested at a temperature of 260 mK with a IMPATT diode radiation source.

## Introduction

Modern superconducting electronics is moving towards lower temperatures, lower noise levels, and higher sensitivity, which can be achieved at sub-Kelvin temperatures using aluminum-based tunnel junctions. One group of devices, such as superconducting quantum interference devices (SQUIDs) and rapid single flux quantum circuits, are based on superconductor–insulator–superconductor (SIS) junctions, another uses superconductor–insulator–normal metal (SIN) junctions. Tunnel junctions based on the SIN structure are widely used, and many different devices are manufactured on their basis [1–3]. These extend from cryogenic thermometers [4–6] and electron coolers [7–10] to various detectors such as Andreev bolometers [11–13], cold electron bolometers [14–15], superconductor–insulator–normal metal–insulator–superconductor (SINIS) bolometers [16–17], and SINIS detectors [18–20]. Here, we present an overview and comparison of our SINIS devices manufactured using different methods. The advantage of Al-based technology is the presence of the intrinsic oxide on its surface, which prevents short circuits with subsequent conductive layers and ensures ease of manufacturing a tunnel barrier, in contrast to Nb-based technology, which requires the formation of an artificial anodic oxide or an additional insulating layer to prevent short circuits, as well as an additional Al layer to form an AlO*_x_* or AlN tunnel barrier.

## Results

### NIS tunnel junction

In tunnel structures, the barrier is a dielectric layer between two metal films (often the oxide layer on the surface of the first metal layer is used as a dielectric). The first experimental study of a tunnel junction was carried out in 1960 [21] for an aluminum–aluminum oxide–lead contact. When tunneling from a normal metal into a superconductor, due to the presence of an energy gap (Δ), only electrons whose energy exceeds Δ can tunnel into the superconductor. Without applying an external voltage or in the case when *eV* < Δ (*T* = 0), tunneling does not occur. Accordingly, a tunnel current occurs when *eV* > Δ. In the case when *T* ≠ 0 the *I*–*V* curve will be smeared [22].

The current–voltage characteristic of a tunnel NIS junction is determined by the following formula [23]:


[1] $$I=\frac{1}{eR_{\text{n}}}\int_{-\infty }^{+\infty }N_{\text{S}}\left(E\right)\left[n_{\text{N}}\left(E-eV\right)-n_{\text{S}}\left(E\right)\right]\text{d}E,$$

where *R*_n_ is the asymptotic resistance of the tunnel junction, *N*_S_(*E*) is the density of states in the superconductor, *n*_S_ is the distribution function in the superconductor, and *n*_N_ is the distribution function in the normal metal. At temperatures *T* ≪ *T*_c_ (*T*_c_ is the critical temperature of the superconductor), the relationship between the tunnel current and voltage can be written using a simplified formula:


[2] $$I=\frac{2\Delta }{eR_{\text{n}}}\sqrt{2\pi k_{\text{B}}T\Delta }\cdot \exp\left(-\frac{\Delta }{k_{\text{B}}T}\right)\sinh\left(\frac{eV}{k_{\text{B}}T}\right),$$

where Δ is the energy gap of the superconductor and *k*_B_ is the Boltzmann constant. The differential resistance is expressed by the following formula:


[3] $$R_{d}=R_{\text{n}}\sqrt{\frac{k_{\text{B}}T}{2\pi \Delta }}\cdot \exp\left(\frac{\Delta }{k_{\text{B}}T}\right){\left(\cosh\left(\frac{eV}{k_{\text{B}}T}\right)\right)}^{-1}.$$

It should also be noted that, in addition to the tunnel current in NIS structures, the presence of a subgap (Andreev) current caused by the Andreev reflection effect is possible [24]. In general terms, the Andreev reflection process is as follows: An electron from a normal metal hitting the SN boundary, passes into the superconductor in the form of a Cooper pair, and a hole is reflected from the SN boundary back into the normal metal. In the simplest case, the Andreev current can be written as the sum of contributions associated with the transfer of electrons in the normal metal (*I*_N_) and the superconductor (*I*_S_), [25]:


[4]

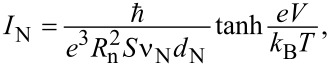




[5]

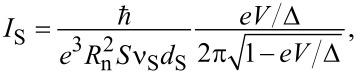



where ν_N_ and ν_S_ are the densities of electron states in a normal metal and a superconductor, *S* is the area of the tunnel NIS junction, *d*_N_ and *d*_S_ are the thicknesses of the normal metal and superconductor films, respectively.

Another feature of the tunnel–NIS junction is the phenomenon of electron cooling. This process is a transfer of heat from a normal metal to a superconductor and is caused by the fact that charge carriers with higher energy (exceeding the value of Δ − *eV*) move from the metal to the superconductor, and those with lower energy remain in the normal metal. As a result, a heat flow arises from the normal metal (*P* is the power of electron cooling) [26]:


[6] $$P\;=\;Q\;-\;IV\;=\frac{1}{e^{2}R_{\text{n}}}\int_{-\infty }^{+\infty }EN_{\text{S}}\left(E\right)\left[n_{\text{N}}\left(E\;-\;eV\right)\;-\;n_{\text{S}}\left(E\right)\right]\text{d}E,$$

where *Q* is the total power released in the superconductor, *IV* is the Joule heat, *n*_N_ and *n*_S_ are the distribution functions of electrons and holes in normal metal and superconductor, respectively. As can be seen from the formula, the electron cooling power depends on both the temperature of the normal metal and the temperature of the superconductor.

### Fabrication techniques

**Figure 1:**
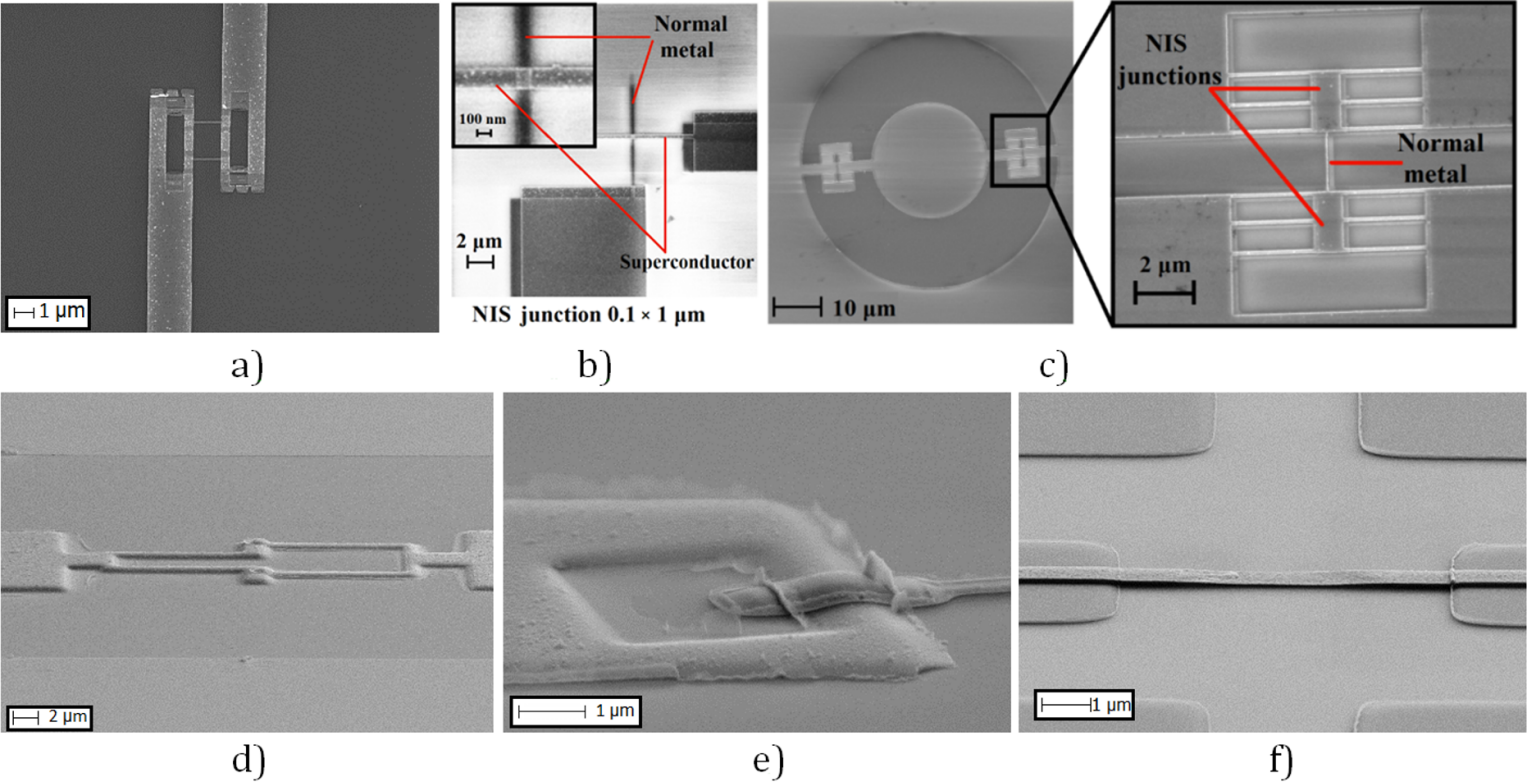
Photos of various tunnel structures fabricated using different technologies. (a) SINIS detector made by using Dolan technology, (b) single tunnel NIS junction and (c) circular antenna with SINIS detector made by Manhattan technology, (d) single SQUID and (e) single NIS junction made by magnetron sputtering with separate lithography, and (f) single SINIS detector with suspended Hf absorber.

Historically, aluminum tunnel junctions were fabricated by shadow evaporation at different angles by the so-called Dolan technology [27]. It requires the formation of a two-layer resist with suspended bridge of the top resist layer (Figure 1a). The advantage of the technique is its simplicity, drawbacks are not very high reproducibility and stability. Another fabrication method is the Manhattan technology [28] with deep orthogonal groves in the resist, see Figure 1b,c. Both methods are based on thermal evaporation at different angles and rotation of substrate, requiring rather sophisticated and expensive deposition plants with thermal or e-beam evaporation. A much more available and simple deposition equipment is magnetron sputtering, but it provides only isotropic deposition, which is incompatible with anisotropic shadow evaporation. The practical solution for a magnetron sputtering is selective etching of different layers of superconducting aluminum and normal metal (e.g., copper). In case of chemical wet etching, this is achieved through an alkali and acid pair, for dry etching, chlorine and fluorine plasmas are used. Alternatively, in the case of magnetron sputtering and separate lithography, ion etching is used before making of the insulator and sputtering of the normal metal (for details see [29]). An example of such a method is presented in Figure 1d,e. Besides SINIS structures with N-absorber on the substrate, we also developed devices with the absorber suspended above the substrate (Figure 1f, technology with wet etching) that are promising for detectors and electron coolers. More details on various technologies and specific features of the fabrication of tunnel structures are provided in a review publication [29].

### Aharonov–Bohm interferometer

**Figure 2:**
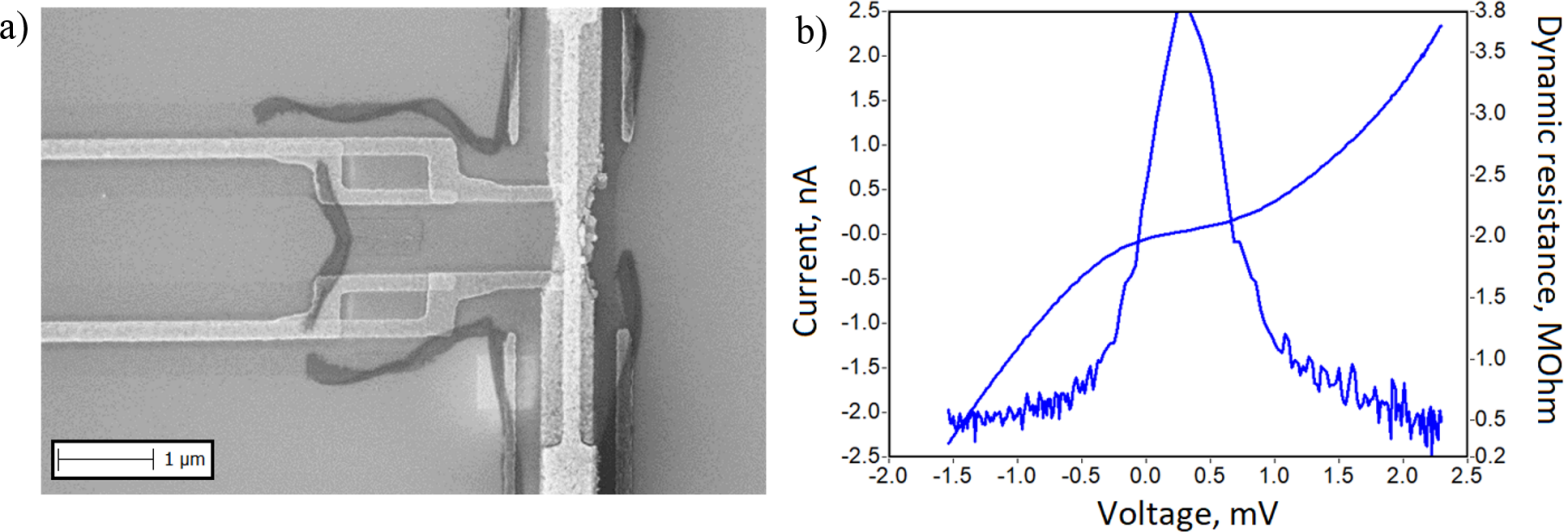
Aharonov–Bohm structure. (а) SEM image of the sample made by Manhattan-type SINIS fabrication and (b) *I*–*V* curve and dynamic resistance of the Aharonov–Bohm interferometer at 330 mK.

The Aharonov–Bohm interferometer is a hybrid nanostructure consisting of a T-shaped normal metal electrode (copper), an insulating tunnel layer (aluminum oxide), and a superconducting fork (aluminum), Figure 2a. Tunnel junction size is 0.2 × 0.2 µm, loop area 2, 4, 8, or 10 µm^2^. The transport characteristics of the fabricated interferometers were studied in sorption ^3^He cryostat at 0.33 K, Figure 2b. The relatively low value of the resistance ratio (*R*_d_/*R*_n_ – dynamic resistance/normal resistance) is associated with the high sensitivity of the interferometer to external noise.

### NIS thermometers

**Figure 3:**
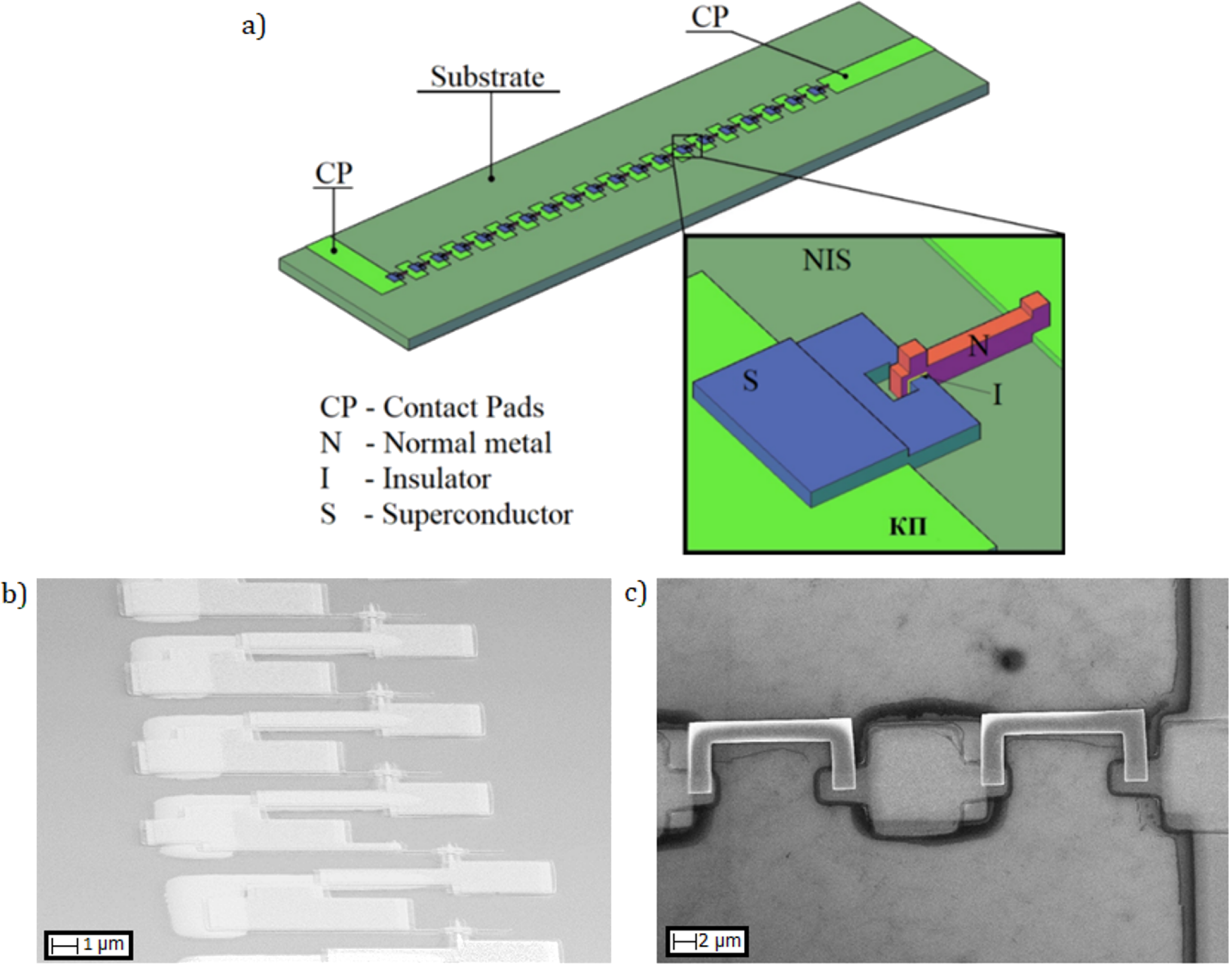
NIS thermometer series array. (a) Schematic view of a thermometer with a chain of 20 NIS junctions and SEM images of thermometers made by (b) Manhattan technology and (c) magnetron sputtering with separate lithography technology.

**Figure 4:**
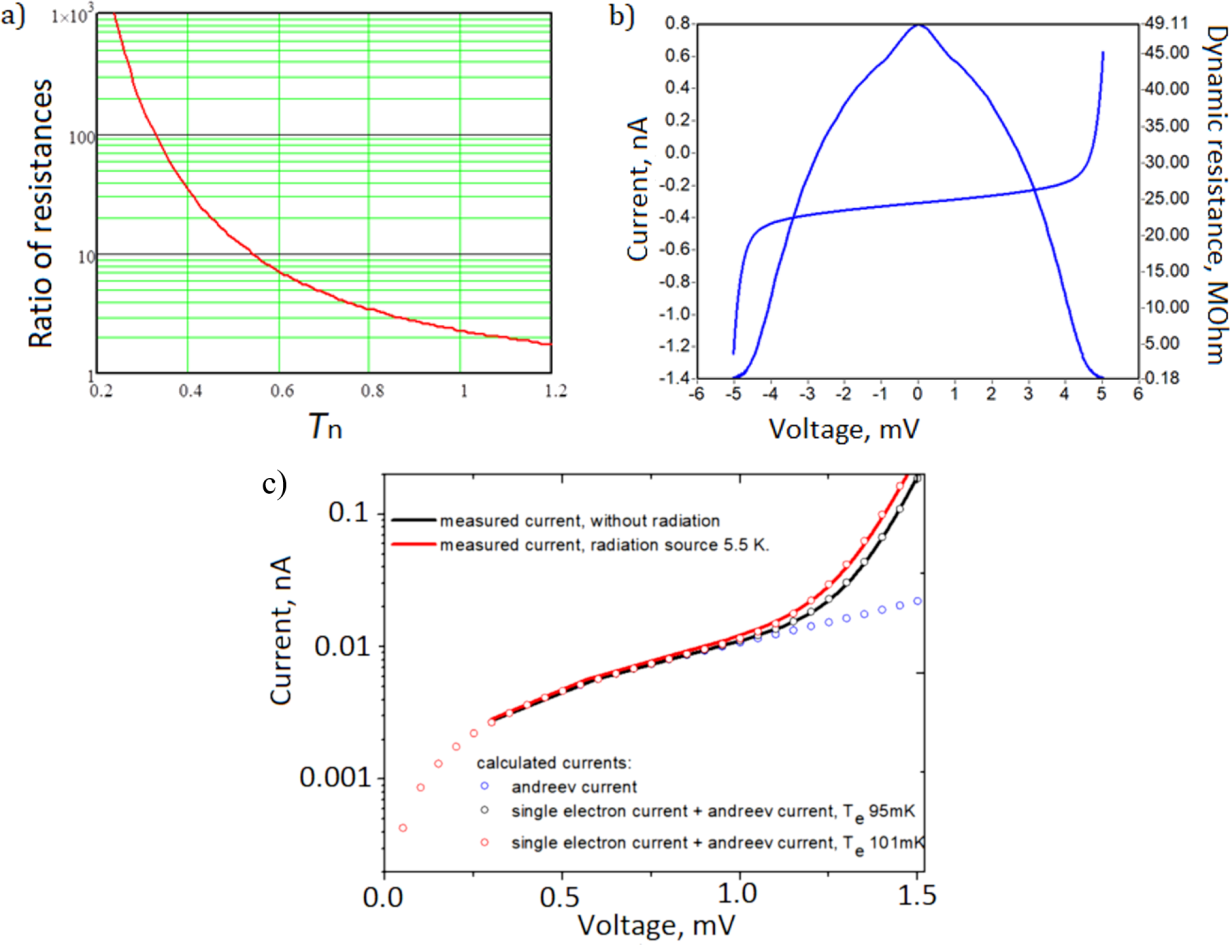
(a) Ratio of resistance at zero bias to the asymptotic resistance using Equation 7, obtained from the general relation in Equation 3. (b) *I*–*V* curve and dynamic resistance for the SINIS thermometer. (c) Measured current with and without radiation. The resistance ratio is *R*_d_/*R*_n_ = 15000 at 95 mK and does not respond to radiation at 5.5 K.

The *I*–*V* characteristic and dynamic resistance of the NIS junction are described by simple relations (Equation 2 and Equation 3), which do not contain fit parameters. In the case of negligibly small Andreev currents suppressed by the ferromagnetic sublayer, such characteristics can be used as primary thermometers [30]. A simple figure of merit for NIS thermometers is the ratio of the resistance at zero bias to the asymptotic one (Equation 7):


[7] $$RR=\left(0.09T_{\text{e}}/T_{\text{c}}\right)\times \text{exp}\left(1.76T_{\text{e}}/T_{\text{c}}\right),$$

obtained from the general relation in Equation 3. For *I*(*V*) = *I*_0_exp[(*eV* − Δ)/*kT*_e_] the temperature sensitivity at low bias current can be approximated as d*V*/d*T*_e_ = (*k*/*e*)ln(*I*/*I*_0_) ≈ −*V*_Δ_/*T*_e_. To reduce the influence of external noise and increase the signal-to-noise ratio, the NIS junctions are connected in series arrays (Figure 3). The temperature dependency of the resistance ratio for aluminum SIN junctions is shown in Figure 4.

### Electron coolers

**Figure 5:**
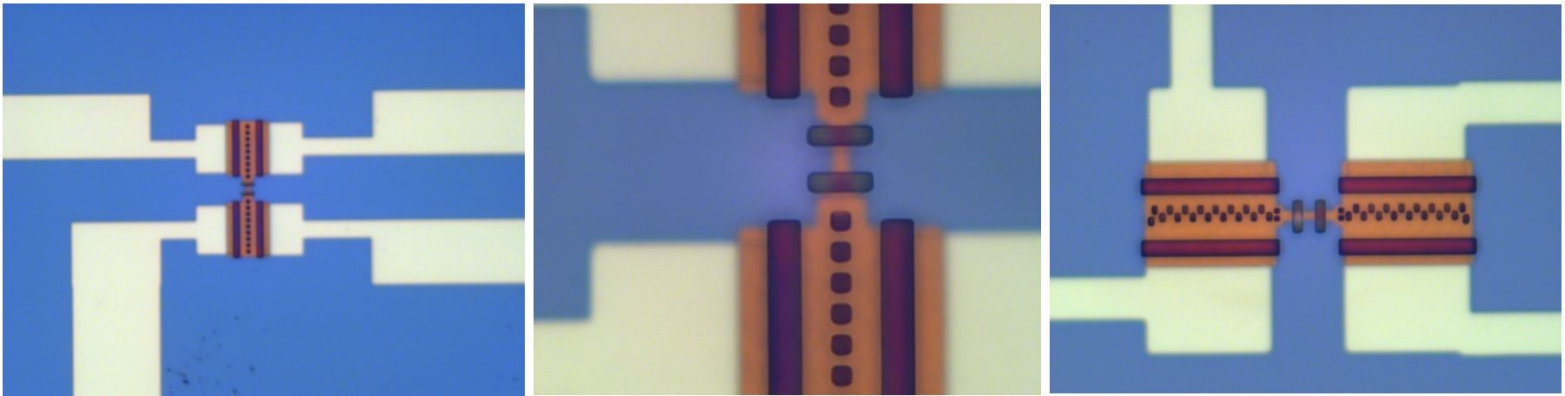
Copper cooler cascade.

**Figure 6:**
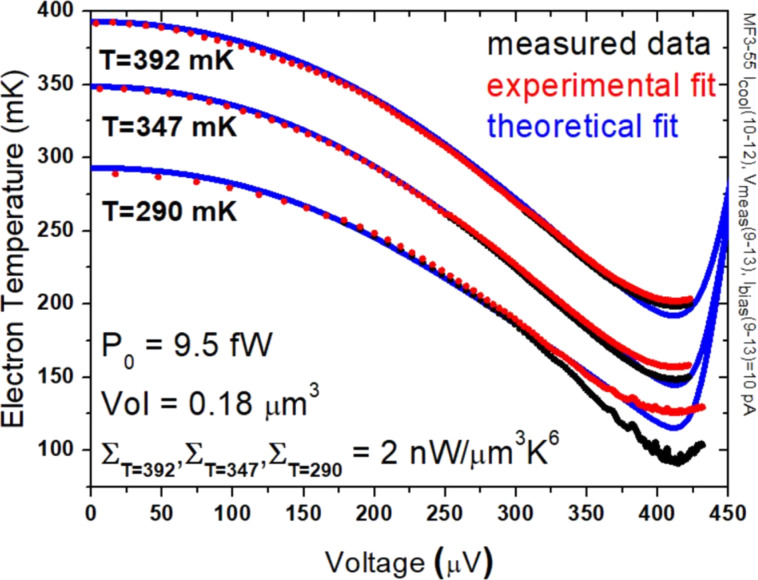
Electron cooling from 392, 347, and 290 mK down to 200, 150, and 95 mK, respectively.

The tunneling current, when biased near the energy gap, carries hot quasiparticles out of the normal electrode, which leads to electron cooling, as in a Peltier element. In a single SINIS structure, it is possible to reduce the electron temperature from 260 to 90 mK [31]. Cascaded NIS coolers can be efficient refrigerators for cooling from 1 K to below 100 mK [32]. One of the problems in explaining results is that, when the injection rate of electrons exceeds the internal relaxation rate in the metal to be cooled, the electrons do not obey the Fermi–Dirac distribution, and the concept of temperature cannot be applied as such. This work is an exciting development towards a fully solid-state, cryogen-free microrefrigerator, which could eventually cover temperatures from the ambient down to the millikelvin range. Figure 5 shows photographs of a two-stage SINIS cooler. The performance of a single-stage SINIS cooler is presented in Figure 6.

### SINIS detectors

The SINIS detector is a thin film of normal metal (the absorber is an element sensitive to incoming radiation) and two NIS junctions that act as a thermometer. An SEM view of the SINIS detector was shown in Figure 1a–f. Often SINIS detectors are considered as classical devices with the optical response equivalent to the electrical response to thermal heating of the absorber by direct current, for example, in [12,14,33–35]. But in practice, the electrical response to heating by direct current is always significantly higher than the optical response for microwave, terahertz, or IR radiation. This is explained by the fact that, in the case of heating the absorber by direct current, the electron temperature of all conduction electrons increases. In the equilibrium state, the electron temperature is determined by the incoming power and the electron–phonon interaction. However, in cases of absorption of a photon with an energy much higher than the thermal energy, the quantum absorption mechanism is already realized, and the real absorption picture becomes much more complicated [19]. It is necessary to take into account many other parameters and relaxation mechanisms. An example of the thermalization process of a radiation quantum with a radiation frequency of 350 GHz is given in [29,36–37]. When an electron absorbs a photon with an energy much higher than the thermal energy, the electron energy will correspond to the electron temperature *hf* = *kT*_e_ of about 16 K for 350 GHz. As a result, a high-energy phonon is created. The process of electron energy relaxation continues until their characteristic times reach the tunneling time determined by the parameters of the SIN transition, that is, the transparency of the barrier, the thickness of the normal metal film, and the diffusion rate of electrons in it. For commonly used structures, this time is of the order of tens of nanoseconds. Typical relaxation times are given in [37–38].

To calculate the sensitivity in the case of heating the absorber with direct current or at low frequencies, the heat balance equation is applicable [12,14]:


[8] $$P_{\text{sig}}+P_{\text{bg}}=\Sigma \Lambda \left(T_{\text{e}}^{5}-T_{\text{ph}}^{5}\right)+P_{\text{cool}},$$

where *P*_sig_ is the signal power, *P*_bg_ is the background radiation power, ΣΛ(*T*_e_^5^ − *T*_ph_^5^) is the heat flux from electrons to phonons, Σ is the material constant, Λ is the absorber volume, *T*_e_ and *T*_ph_ are, respectively, the electron and phonon temperatures of the absorber, and *P*_cool_ is the electron cooling power. In other cases, it is necessary to move on to the analysis of collision integrals and the kinetic equation [19–20].

One of the main characteristics of the detector is the noise equivalent power (NEP) [W·Hz^−1/2^], that is, the power of the useful signal in a unit of frequency band, equivalent to the noise power in the device receiving the signal. Approximately, in the simplest case, the NEP of the SINIS detector can be calculated using the following formula:


[9] $${\text{NEP}}^{2}={\text{NEP}}_{\text{e}-\text{ph}}^{2}+{\text{NEP}}_{\text{NIS}}^{2}+{\text{NEP}}_{\text{amp}}^{2}$$

NEP_e−ph_ is the noise equivalent power of electron–phonon interaction, caused by the discreteness of energy exchange between electrons and phonons:


[10] $${\text{NEP}}_{\text{e}-\text{ph}}=\sqrt{10k_{\text{B}}\Lambda \Sigma \left(T_{\text{e}}^{6}+T_{\text{ph}}^{6}\right)},$$

where *k*_B_ is the Boltzmann constant, Λ is the volume of the absorber, Σ is the constant of electron–phonon interaction, and *T*_e_ and *T*_ph_ are the temperatures of the electron and phonon subsystems, respectively. NEP_NIS_ is the noise equivalent power of NIS junction. It is a combination of shot noise that occurs as a result of the charge transfer by electrons during tunneling through the tunnel SIN junction and the thermal noise of these same electrons as heat carriers:


[11] $${\text{NEP}}_{\text{NIS}}=\sqrt{\frac{\delta I_{\omega }^{2}}{S_{\text{V}}^{2}}{\left(\frac{\text{d}V}{\text{d}I}\right)}^{2}+\delta P_{\omega }^{2}-2\frac{\langle \delta P_{\omega }\delta I_{\omega }\rangle }{S_{\text{V}}\left(i\right)}\frac{\text{d}V}{\text{d}I}}.$$

**Figure 7:**
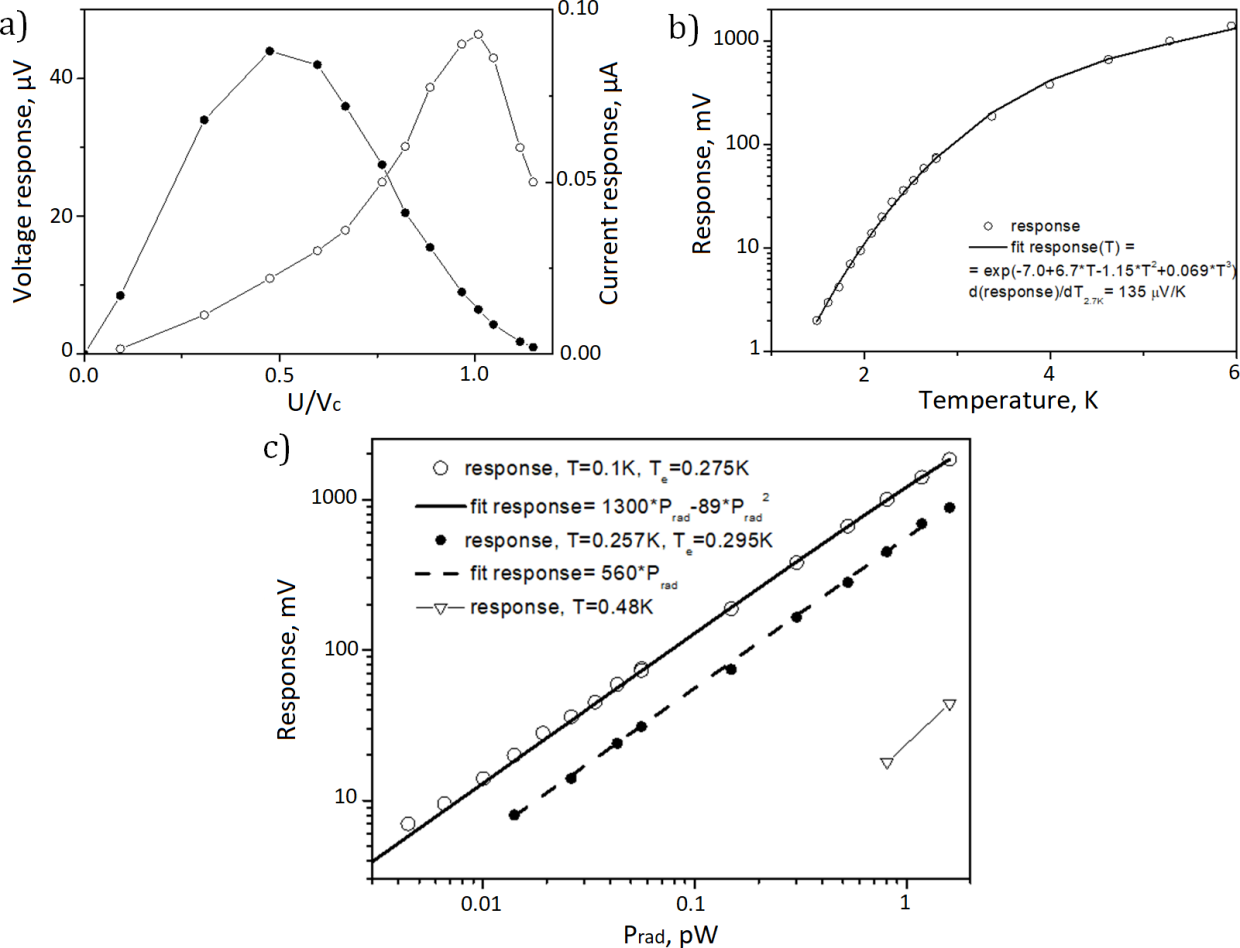
Some of SINIS detectors measured characteristics. (a) Current and voltage response for a parallel array for *P*_rad_ = 1 pW and maximum current responsivity of d*I*/d*P* = 72000 A/W. (b) Voltage response on black body temperature; at 2.7 K, it is d*V*/d*T* = 135 µV·K^−1^, (c) voltage response at temperatures of 0.1, 0.295, and 0.48 K; the maximum response is 3.9 V/nW.

δ*I*_ω_^2^ is the power spectral density (PSD) of current fluctuations due to shot noise, δ*P*_ω_^2^ is the PSD of thermal fluctuations, and ⟨δ*P*_ω_δ*I*_ω_⟩ is the correlation between the two types of noise. NEP_amp_ is the noise equivalent power of the readout amplifiers. We develop typical characteristics of the detectors based on SINIS structures, namely, volt–watt responsivity no worse than 10^9^ V·W^−1^, noise equivalent power below 10^−17^ W·Hz^−1/2^ (the value is given for measurements with a room-temperature JFET readout system and can be improved by upgrading the readout electronics). As an example, some measured characteristics of the SINIS detectors are given in Figure 7. The amplifier voltage noise is 20 nV·Hz^−1/2^, the optimal input impedance for this amplifier is 500 kΩ, and the maximum response is measured at a bias voltage of 0.5 Δ/*e*.

The list of designed and fabricated devices contains different arrays of SINIS detectors for frequencies from below 100 GHz to above a few terahertz, broadband detectors integrated with log-periodic antennas, and narrow-band detectors integrated in twin-slot and double-dipole antennas. The miniature size of SINIS detectors allows them to be integrated into antennas of various types. Also, the detector itself is not frequency-selective, but by integrating such devices into various antennas and using additional filter elements, the detecting device can be tuned to the required frequency. Single antennas are convenient to use for test studies or for use under conditions of low background load and signal. This is due to the fact that the saturation level of a single SINIS detector is 0.5–1.0 pW. Under conditions with a high background load (for example, for ground and balloon observations), such detectors are combined into matrices; the power of incoming radiation is distributed between the matrix elements and, accordingly, the dynamic range of the detecting device can be significantly increased.

### Deployment at “Big Telescope Alt-azimuthal”

Currently, work is underway to implement the SINIS detectors on a practical instrument, that is, the optical observatory “Big Telescope Alt-azimuthal” (BTA SAO RAS) for observations in the range of 75–110 GHz. The choice of an optical observatory is due to the fact that there are no large-scale subterahertz observatories in Russia, but there are agreements and the possibility of conducting research based on the BTA observatory. Estimates of the possibility of observations, a description of the receiving system, and the current status of the work are given in [39–41]. These works will allow not only to conduct full-scale tests of the SINIS detectors, but will also expand the capabilities of the BTA observatory to the subterahertz range and use it not only for observations, but also for testing new technologies of subterahertz detectors.

According to [42], the thermodynamic temperature of the planets of the solar system at a frequency of 100 GHz can be estimated as Mars 198 K, Jupiter 172.6 K, Saturn 145 K, Uranus 121 K, and Neptune 118 K; the brightness temperature of the Moon can vary from 100 to 400 K, depending on the time of day. These values exceed the sky temperature at the zenith for the BTA and can be measured with a signal-to-noise ratio greater than unity by the described receiver even without diagram.

Approximate estimates for observing Jupiter and other planets are made and compared with figures from [42] (central frequency *f*_c_ = 100 GHz, bandwidth 10%, Δ*f* = 10 GHz. The thermodynamic temperature of Jupiter at 100 GHz is 172.6 K; the angular diameter of Jupiter is 41.68 arcsec; the BTA main mirror diameter is 6 m, and the aperture efficiency of 0.8 gives an effective area of *A*_eff_ = 22.6 m^2^. The solid angle occupied by Jupiter is of the order of Ω = 3.20 × 10^−8^ sr. The spectral flux density is *S* = *I* × Ω = 1677 Jy = 1.677 × 10^−23^ W·m^−2^·Hz^−1^. For 172.6 K at 100 GHz, we have a blackbody intensity *I* = 5.23 × 10^−16^ W·m^−2^·Hz^−1^·sr^−1^. The power density collected by a telescope with an effective aperture of 22.6 m^2^ is *P* = 3.79 × 10^−22^ W·Hz^−1^. The total power in the 10% band recorded by the receiver from Jupiter is *P*_tot_ = 3.79 × 10^−12^ W. For planets visible in November 2025 at 100 GHz, the flux densities will be as follows: Jupiter 41.68 arcsec, 172.6 K, 1677.08 Jy; Saturn 18.68 arcsec, 145.7 K, 283.63 Jy; Mars 3.87 arcsec, 192.3 K, 16.13 Jy; Uranus 3.8 arcsec, 120.5 K, 9.67 Jy; and Neptune 2.34 arcsec, 117.4 K, 3.57 Jy.

**Figure 8:**
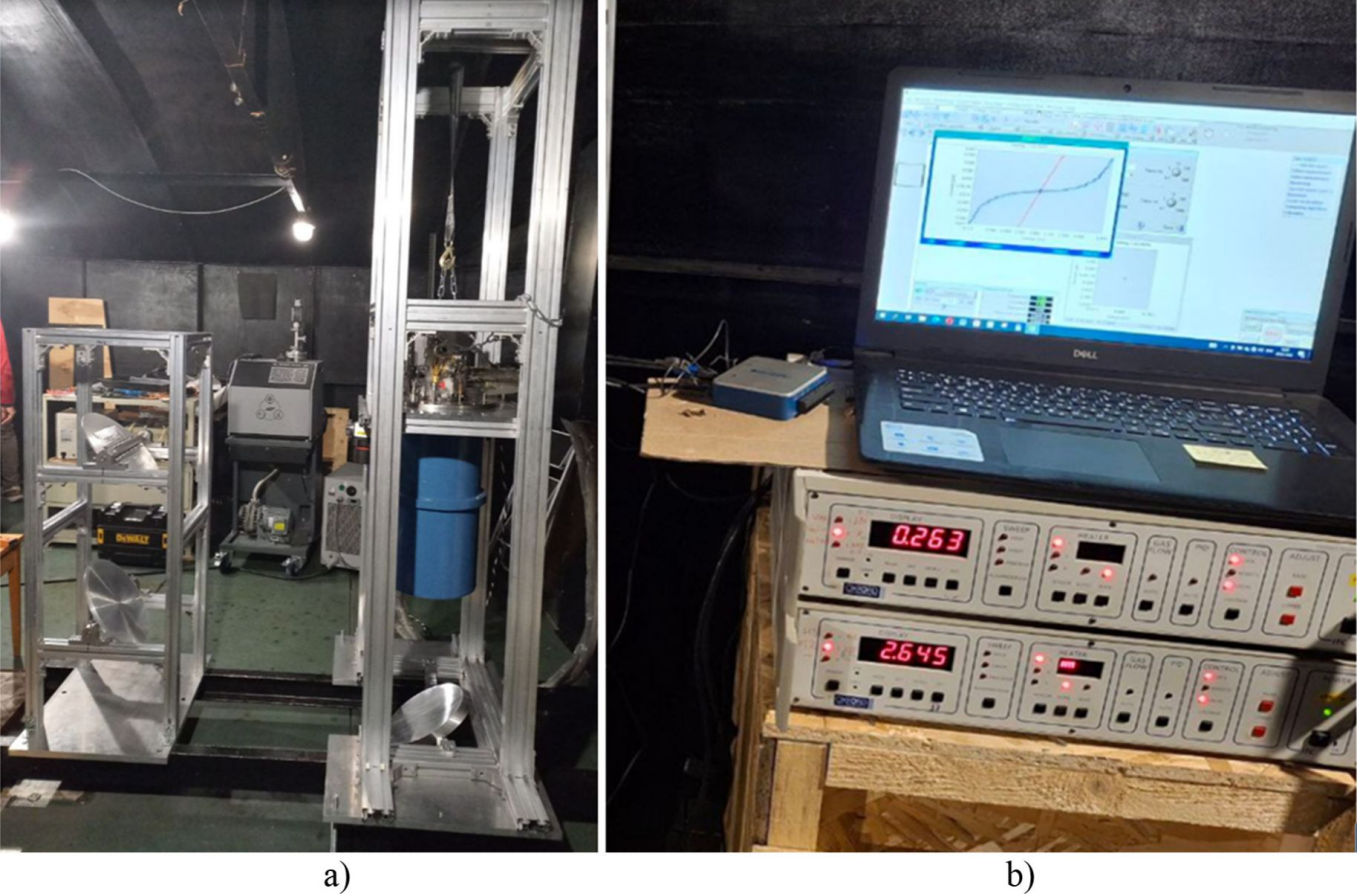
Installation and initial testing of the receiving system based on SINIS detectors at the BTA SAO RAS observatory. (a) Installed racks with a Heliox AC-V cryostat and (b) measured *I*–*V* characteristics of the SINIS detector matrix installed inside the cryostat with a closed optical window and under irradiation by the source; The lower part demonstrates a PT2 temperature of 2.6 K and a ^3^He pot temperature of 263 mK.

At this stage, the necessary equipment and elements of the quasi-optical path in the “Nasmyth 1” cabin of the BTA telescope have been installed. Also, primary tests of the quasi-optical path for focusing incoming radiation and irradiation of the source (IMPATT diode) of the detecting matrix in the 75–110 GHz range were carried out. Photos from the expedition are presented in Figure 8. The ratio of resistances on the presented current–voltage characteristic is about 30, which, according to Equation 3, corresponds to an electron temperature of 0.5 K with a physical temperature of 0.26 K.

**Figure 9:**
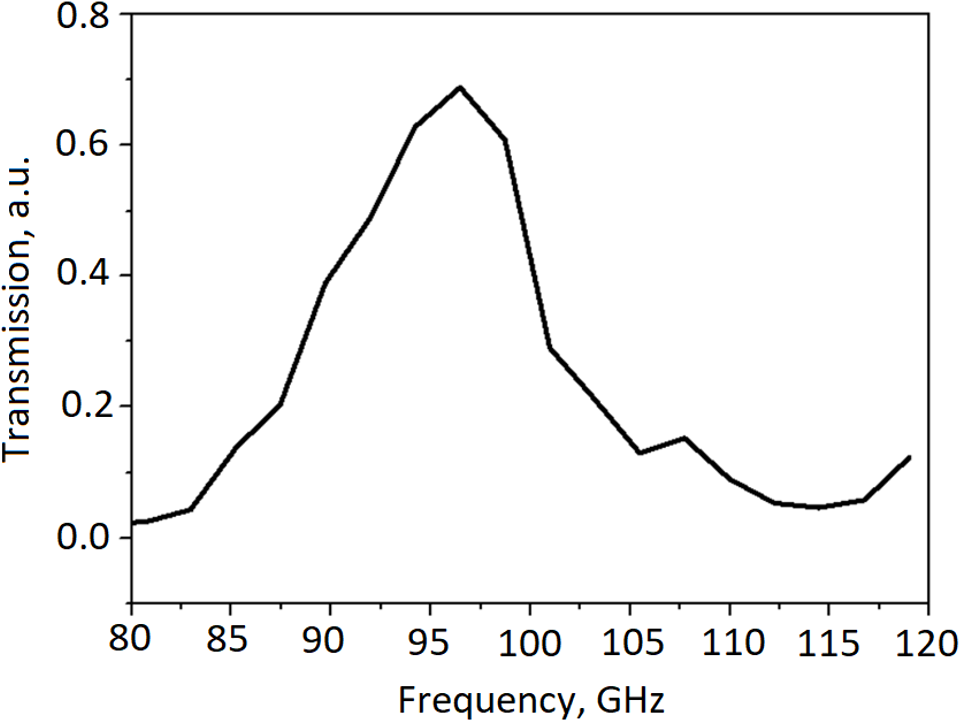
Transmission spectrum of the cold mesh filter.

**Figure 10:**
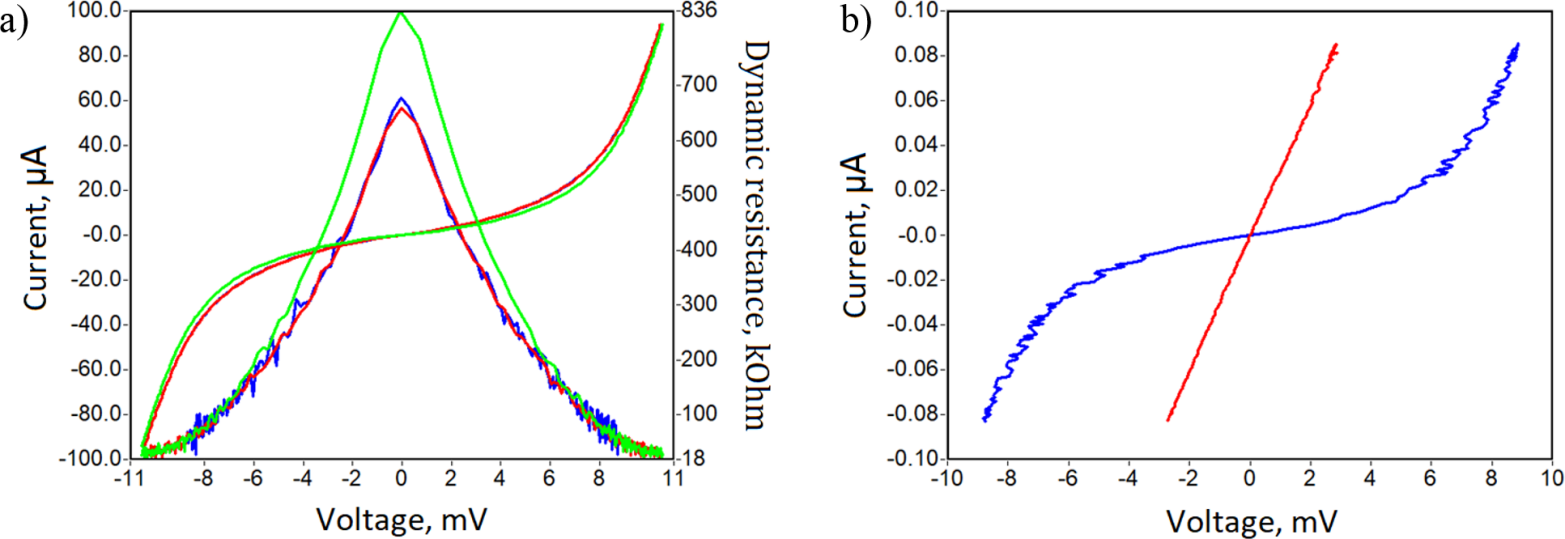
(a) *I*–*V* curves and dynamic resistance of series SINIS array and (b) *I*–*V* curve without (blue) and with (red) microwave radiation at 95 GHz.

The array structure was mounted in back-to-back horn matching structure with an optimum for 95 GHz and corresponding quasi-optical band-pass filters (Figure 9). *I*–*V* curves and dynamic resistances are presented in Figure 10a. The resistance ratio is 46, which corresponds to an electron temperature of about 0.36 K. In these measurements, there was no optical window and no strong overheating of the sample by radiation.

## Discussion

We have designed, fabricated, and experimentally studied a family of aluminum SINIS devices. Microwave detectors at 100 mK demonstrated responsivity up to 10^9^ V·W^−1^, array of NIS thermometers provide sensitivity down to 10 µK, electron coolers can reduce the electron temperature of a normal metal absorber from 280 mK down to 100 mK, and the SINIS receiver for a frequency of 95 GHz was installed on the BTA telescope and tested at a temperature of 260 mK.

## Data Availability

Data generated and analyzed during this study is available from the corresponding author upon reasonable request.
